# Supplementary material for: Postpartum Depression in COVID-19 Days: Longitudinal Study of Risk and Protective Factors
Source: J Clin Med. 2022 Jun 17;11(12):3488. doi: 10.3390/jcm11123488 (PMC9224599; doi:10.3390/jcm11123488)
Supplement: Supplementary file 1 [file jcm-11-03488-s001.zip › jcm-1695420-supplementary.pdf]

Supplementary S1. QUESTIONNARE FORM

Hello,

In the following survey you will find questions regarding your current birth experience and your postpartum status. The questionnaire is important in order for us to understand the consequences of childbirth and postpartum in times of the Coronavirus.

The participation in this research is voluntary. You may stop the survey at any given moment.

The privacy of your answers is guaranteed, and publications of study results will be performed anonymously with no personal details of participants.

The filling out of this survey will be considered as an informed consent for participating in this research.

Thank you in advance for your cooperation!

1. Please fill in the personal code you received from us _______

<u>DEMOGRAPHICS</u>

1. Age
2. Gender (woman, other)
3. Country of birth (Israel, other)
4. Religion (Judaism, Islam, Christianity, not religious, other)
5. Marital status (single, in an unmarried relationship, married, divorced, separated, widowed, other)
6. Family type (heterosexual couple, homosexual couple, single parent, other)
7. Educational level (primary, high school graduate, first degree, second degree, third degree and up)

8. Average household income (the current average household income in Israel is 19,500 NIS) (much below average, below average, average, above average, much above average)
9. Have you gone through any traumatic events in the last 6 months? (e.g car accident, death of a loved one, etc')? if so, what was the traumatic event?
10. What is your profession?
11. Current occupational status (on unpaid leave due to the Coronavirus, on unpaid leave by choice, on maternity leave, working part-time, working full-time, other)
12. What is your partner's profession?
13. Current occupational status of partner (on unpaid leave due to the Coronavirus, on unpaid leave by choice, on birth leave, working part-time, working full-time, other)
14. Have you experienced any financial difficulties due to the Coronavirus?
15. How often do you worry about your financial status due to the Coronavirus? (not at all, almost never, sometimes, almost all the time, all the time)
16. Do you have any background medical conditions which put you at risk in case of being infected with the Coronavirus? (yes, no) if yes- what is your medical condition?
17. Do you have any physical, cognitive or mental (diagnosed or undiagnosed) disability, such as ADHD, depression, autism, deafness, chronic medical conditions? (yes, no), if yes- what is your disability?

COVID-19 RELATED LIFE EVENTS

Here is a list of events that you may have encountered in the last few months following the outbreak of the COVID-19 pandemic. Please select at each event whether you experienced it or not.

	Have I experienced such an event	If so - how hard was it for me
1. I was in contact with someone who was placed in quarantine (before being placed in quarantine)	Yes/ No	Not difficult/ Somewhat difficult/ Extremely difficult
2. I was in contact with someone infected with COVID-19	Yes/ No	Not difficult/ Somewhat difficult/ Extremely difficult
3. Someone close to me (other than family member) was placed in quarantine	Yes/ No	Not difficult/ Somewhat difficult/ Extremely difficult
4. Someone close to me (other than family member) got infected with COVID-19	Yes/ No	Not difficult/ Somewhat difficult/ Extremely difficult
5. One of my relatives was placed in quarantine	Yes/ No	Not difficult/ Somewhat difficult/ Extremely difficult
6. One of my relatives got infected with COVID-19	Yes/ No	Not difficult/ Somewhat difficult/ Extremely difficult

7. I was placed in quarantine	Yes/ No	Not difficult/ Somewhat difficult/ Extremely difficult
8. I was infected with COVID-19	Yes/ No	Not difficult/ Somewhat difficult/ Extremely difficult
9. Someone I know died as a result of COVID-19	Yes/ No	Not difficult/ Somewhat difficult/ Extremely difficult
10. One of my relatives died as a result of COVID-19	Yes/ No	Not difficult/ Somewhat difficult/ Extremely difficult
11. My children stayed at home as a result of the education system shutdown	Yes/ No	Not difficult/ Somewhat difficult/ Extremely difficult
12. I refrained from meeting family members as a result of the Ministry of Health guidelines	Yes/ No	Not difficult/ Somewhat difficult/ Extremely difficult
13. I refrained from meeting family members as a result of fear of infection	Yes/ No	Not difficult/ Somewhat difficult/ Extremely difficult
14. Family members stopped helping me because of fear of infection	Yes/ No	Not difficult/ Somewhat difficult/ Extremely difficult

FEAR OF COVID-19

How much do you agree with the following statements?

	Definitely disagree	Disagree	Does not agree and does not deny	Agree	Definitely agree
1. I am most afraid of COVID-19					
2. It makes me uncomfortable to think about COVID-19					
3. My hands become clammy when I think about COVID-19					
4. I am afraid of losing my life because of COVID-19					
5. When watching news and stories about COVID-19 on social media, I become nervous or anxious					
6. I cannot sleep because I'm worrying about getting COVID-19					

7. My heart races or palpitates when I think about getting COVID-19					

Edinburgh Postnatal Depression Scale (EPDS)

Please enclose in each group the sentence that best describes your feelings in the past month, including today.

In the last past month:

1. I have been able to laugh and see the funny side of things
    a. As much as I always could
    b. Not quite as much now
    c. Definitely not so much now
    d. Not at all

2. I have looked forward with enjoyment to things
    a. As much as I ever did
    b. Rather less than I used to
    c. Definitely less than I used to
    d. Hardly at all

3. I have blamed myself unnecessarily when things went wrong
    a. Yes, most of the time
    b. Yes, some of the time
    c. Not very often
    d. No, never

4. I have been anxious or worried for no good reason
    a. No, not at all

   b. Hardly ever
   c. Yes, sometimes
   d. Yes, very often

5. I have felt scared or panicky for no very good reason
   a. Yes, quite a lot
   b. Yes, sometimes
   c. No, not much
   d. No, not at all

6. Things have been getting on top of me
   a. Yes, most of the time I haven't been able to cope at all.
   b. Yes, sometimes I haven't been coping as well as usual
   c. No, most of the time I have coped quite well.
   d. No, I have been coping as well as ever.

7. I have been so unhappy that I have had difficulty sleeping
   a. Yes, most of the time
   b. Yes, sometimes
   c. Not very often
   d. No, not at all

8. I have felt sad or miserable
   a. Yes, most of the time
   b. Yes, quite often
   c. Not very often
   d. No, not at all

9. I have been so unhappy that I have been crying

a. Yes, most of the time
b. Yes, quite often
c. Only occasionally
d. No, never

10. The thought of harming myself has occurred to me
    a. Yes, quite often
    b. Sometimes
    c. Hardly ever
    d. Never

The State-Trait Anxiety Inventory - STAI

	Not at all	A little	Somewhat	Very much so
I feel calm	1	2	3	4
I feel tense	1	2	3	4
I fell upset	1	2	3	4
I am relaxed	1	2	3	4
I feel content	1	2	3	4
I am worried	1	2	3	4

MSPSS

		Very strongly disagree	Strongly Disagree	Mildly Disagree	Neutral	Mildly Agree	Strongly Agree	Very Strongly Agree
1	There is a special	1	2	3	4	5	6	7

	person who is around when I am in need							
2	There is a special person with whom I can share joys and sorrows	1	2	3	4	5	6	7
3	My family really tries to help me	1	2	3	4	5	6	7
4	I get the emotional help & support I need from my family	1	2	3	4	5	6	7
5	I have a special person who is a real source of comfort to me	1	2	3	4	5	6	7
6	My friends really try to help me	1	2	3	4	5	6	7

7	I can count on my friends when things go wrong	1	2	3	4	5	6	7
8	I can talk about my problems with my family	1	2	3	4	5	6	7
9	I have friends with whom I can share my joys and sorrows	1	2	3	4	5	6	7
10	There is a special person in my life who cares about my feelings	1	2	3	4	5	6	7
11	My family is willing to help me make decisions	1	2	3	4	5	6	7
12	I can talk about my	1	2	3	4	5	6	7

	problems with my friends							
